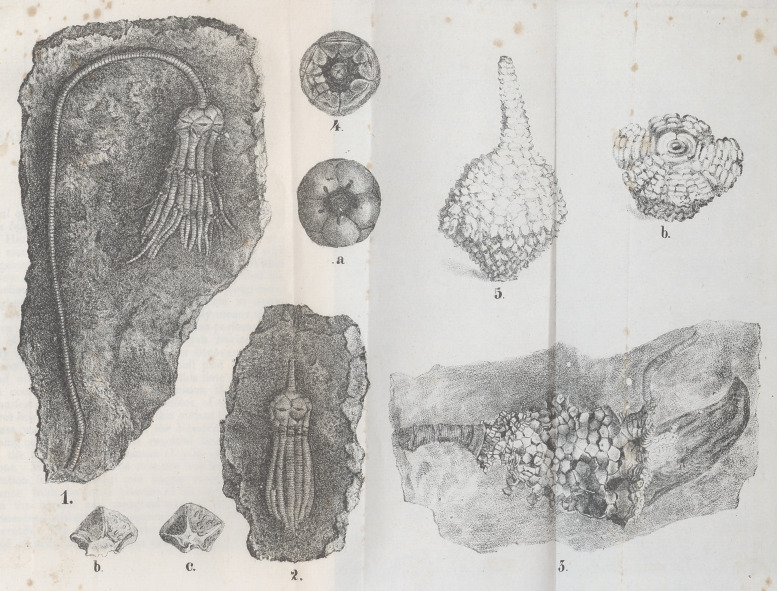# Contributions to the Geology of Kentucky

**Published:** 1847-10

**Authors:** Lunsford P. Yandell, Benj. F. Shumard


ART. III.—*Contributions to the Geology of Kentucky.* By LUNSFORD P. YANDELL, M.D., and BENJ. F. SHUMARD, M.D.

When we first thought of preparing the following paper, we proposed limiting our remarks to the Geology of the country in the immediate neighborhood of the Falls of the Ohio, the fossils of which we have been several years engaged in collecting; but having made, some months since, an excursion into the interior of the State as far as the Grayson Springs, we have concluded to give to our communication a wider range. Our researches have been directed especially to the character and relative age of our rocks as exhibited in their organic remains, and by the list of these, which we shall be able to present, identical with European species, we hope that we shall be found to have contributed something towards establishing certain important points in geology. The parallelism of the formations of America and Europe, is a subject which just now is engaging the attention of some of the first geologists of the age. It is interesting, moreover, to compare the formations of different and distant portions of our own continent. In order to this, many observations must be recorded. The field in which we have pursued our inquiries is one of exceeding interest, and the facts which we are about to communicate, as the result of our researches, it is hoped, will not be without their value in settling some of the great principles of geological science.

In our account of the formations of Kentucky, we shall commence with the lowest strata and review the formations as they present themselves in the ascending order.

On the Indiana side of the Falls of the Ohio, at extreme low water, the *Silurian* strata make their appearance, and for the distance of eight miles in a direction nearly south form the surface rocks. They are for the most part of a light greyish color, compact and durable, affording an excellent material for building purposes, for which they are quarried extensively at a number of places in the neighborhood of Louisville. On Bear-grass creek, a mile to the east of the city, the lowermost layers of this formation to be seen in this vicinity, are exposed; and here we find in considerable abundance a *Pentamerus*, resembling very closely Hall's figure in the New York Geological reports of *P. oblongus*, from the Clinton group of New York, and answering well to the description and figure, in Murchison's Silurian system, of the same fossil from the upper beds of the Caradoc formation in England. If our fossil be really identical in species, of which we entertain no doubt, then the rocks in question may be referred to the upper portion of the lower Silurian system; and may be considered as the western representative of the *Clinton group* of New York, and the *Carodoc* of England.

The coralline beds of the Magnesian limestone of Iowa and Wisconsin, and also those of Ohio and Indiana, contain numerous internal casts of a *Pentamerus*, which are probably referable to this species;* but until more perfect specimens are found it will be difficult to decide with certainty in regard to their identity.

Associated with the above we sometimes find a very large *Orthoceratite*, the species of which is yet undetermined. Professor Cobb has a very perfect specimen which he obtained

---

*See D. D. Owen's Geological report of Iowa, Wisconsin, and Illinois, page 78, pl. 14, fig. 40.

from these rocks near Utica, Indiana. In length it measures upwards of two feet. We also saw in Perry county, Tennessee, this multilocular shell of the same length, but here it was so firmly imbedded in the rocks that it could not be detached.

We have lately found in these strata on Bear-Grass creek very perfect specimens of *Caryocrinus ornatus*, differing from the New York specimens of this encrinite in being more elongated, and having its external markings better defined. In the same connexion we also find *Calymene Blumenbachii*, and several undetermined species of encrinites.

Considered in their lithological characters, our Pentamerus beds correspond more closely with those of New York than with those of Iowa, Wisconsin, and Ohio, the former being of a shaly, argillaceous character, while the latter are invariably magnesian.

Immediately above these beds occur a series of strata varying from twenty to thirty feet in thickness, remarkable for the number of fossils, principally *Polyparia*, contained in them. Of these the most characteristic is *Catenipora escharoides* and *Cyathophyllum dianthus*. The first is the most abundant coral found here, and is distributed through all the layers, though the upper ones contain it in the greatest abundance and perfection. The coral itself is silicious, while its matrix is calcareous, in conseqence of which the latter disintegrates readily, leaving the former beautifully preserved on the surface of the rocks. The chain-coral seems to have a wider vertical range than any other fossil found in our formations. It occurs in the upper Silurian rocks, near Dayton, Ohio, as on our Falls, and in the glades of Perry county, Tennessee, and the Magnesian beds of Iowa and Wisconsin. Dr. Troost, in his geological reports of Tennessee, mentions *C. labyrinthica*, which is now considered as merely a variety of *C. escharoides*, as occurring at Eddyville, Kentucky, in rocks which belong to the carboniferous limestone; and Dr. Clapp has found it as high up as the coal series.

*Cyathophyllum dianthus*, of Hall, is also occasionally met with. In New York this fossil occurs in the Onondaga limestone, a group which seems to hold a higher position than those under consideration. It is silicious in its character.

A number of corals are associated with the above, but for the want of proper works of reference they have not yet been satisfactorily determined. The descriptions and figures of *Strombodes helianthoides* and *Stromatopora polymorpha* given by Goldfuss, correspond very closely with two species from this locality, and in addition to these, a number of forms belonging to the genera *Strombodes*, *Cyathophyllum*, *Favosites*, *Retipora*, and *Syringapora*, are of frequent occurrence. A few varieties only of shells characterize these strata, of which two species have been identified; viz: *Terebratula Wilsoni*, and *T. reticularis*. In the glades of Perry county, Tennessee, we have also found these shells associated with *Pentremites Reinwardti*, *Caryocrinus ornatus*, and *Pentamerus galeatus*.

The foregoing are some of the most characteristic fossils which have come under our notice in our Silurian rocks, but future investigations will no doubt bring to light many other forms with which we are now unacquainted.

By reference to table I, at the end of this paper, it will be seen that many of the fossils just mentioned are identical with species figured by Hall from the Niagara group of New York; and consequently it appears that our strata on Bear-Grass, above the Pentamerus beds, and the lowermost layers on the Falls are the western equivalents of that group. The limestone of Perry county, Tennessee, containing *Caryocrinus ornatus*, *Terebrat. Wilsoni*, *T. reticularis*, and *Pentremites Reinwardti* may also, on strong palæontological grounds, be referred to the same geological age.

Immediately above these rocks a series of strata occur remarkable for the abundance of their fossils. The beds of this formation have been divided by Dr. Clapp into 1st, Lower or Coralline beds; 2d, Middle or Shell beds; and 3d,

Upper or Limestone beds. The Coralline beds rest immediately upon the above mentioned Silurian strata, and are seen to the best advantage on the Falls of the Ohio at extreme low water, at Charlestown landing, on the Ohio river, twelve miles above Louisville, and in Floyd county, Indiana, eight miles northeast of New Albany. At these localities *polyparia* occur in the greatest profusion and perfection, immense beds being made up almost exclusively of these forms. The laminæ of the rocks separate easily on the planes of deposition, so that good specimens of these fossils may be extricated without much difficulty. The following are some of them:

*Favosites Gothlandica.*—At each of these localities, and in fact wherever these rocks are exposed, this fossil is found in abundance, but the point where it occurs in the greatest perfection is Goose island, near Louisville, where it is met with of a snowy whiteness. Dr. Clapp makes a new species of the coral in which two, three, and even four rows of pores are displayed in the same specimen. F. Gothlandica has a very wide geographical distribution. In the United States, it occurs in Tennessee, Missouri, Kentucky, Ohio, and New York, and in Europe it is found at St Petersburg, Eifel, Aymestry, Wenlock, and a number of other localities.

*F. hemispherica.*—This fossil is abundant on the Falls, and is found in masses of a hemispherical figure, which may vary from one to ten inches in diameter. It is most commonly calcareous, though sometimes it is silicious.

*F. maxima.* (Troost).—This coral, associated with the above, also occurs abundantly on the Falls of Ohio. It is silicious or silico-calcareous. It also occurs in Floyd county, Indiana, and in Iowa and Wisconsin.

*F. polymorpha.*—The best locality for this coral is Goose island, where it may be obtained in great quantities, varying from one line to four inches in diameter, and a foot or more in length. It is also found in Ohio, New York, Iowa, and Wisconsin. In Europe it occurs at Ludlow and Esthonia, in Silurian, while at Eifel, Plymouth, and Paffrath it is found in

Devonian strata. The coralline beds contain also *Favosites spongites*, and *F. basaltica*.

*Astrea rugosa.*—A coral, identical in species with one figured by Hall in the New York Geological reports under this name, is found on the Falls, and at Charlestown landing. It is also found in Iowa in great masses, where it is commonly known as the Iowa marble. (See review of N. York Geological reports in Silliman's Journal, 2d series, vol. 1, p. 60, fig. 2).

*Syringapora tubiporoides.*—This beautiful coral is rare. The only specimens we have seen were obtained from the Falls. It is always silicious, and what is extraordinary, is almost invariably found incrusting other corals, a very fine specimen in our possession having for its nucleus the Cystiphyllum vesiculosum, and another completely investing a Cyathophyllum. Three undetermined species of the genus Syringapora also occur in the coralline beds, which like *S. tubiporoides* are generally silicious, and one of which, judging from the figure in Hall's report (p. 160, pl. 63, fig. 3), is identical with a species from the Onondaga limestone of New York. It is of rare occurrence on the Falls, but in Clark county, Indiana, on Bear-Grass, and in Bullitt county, Kentucky, it is more abundant, occurring at the latter locality in masses of fifty pounds weight. This is a laminiferous coral, however, and not properly a Syringapora.

*Cyathophyllum gigas.* (Clapp).—This is a very common and characteristic fossil, abounding at all the localities where the coralline strata are exposed, and on the Falls, frequently affording specimens two feet in length. It is both silicious and calcareous; the septa in the former case are often incrusted with perfectly formed crystals of quartz, which give to the specimens, when broken, an exceedingly beautiful appearance.

The following, as well as a number of undetermined species of this genus, are associated with the above; viz: *C. punctatum* and *C. scabrum.* Another species is occasionally met with which closely resembles *C. cæspitosum* of Goldfuss.

This is very far from being a full list of the corals yielded by the beds of which we have been speaking, but the others, if not undescribed, are at least unknown to western geologists.

The species of shells in the coralline beds are not numerous, and for the most part are limited to a single stratum; one species, however, abounds—the *Delthyris gregaria* of Clapp, which bears a strong resemblance to the *Delthyris zigzag* of Hall, from the Hamilton group of New York. Perfect specimens of this shell are not often procured. Associated with it, several undescribed species of *Gasteropoda* occur, one of which, a *Tarbo*, is nearly five inches in diameter. This shell is generally silicified, and it not unfrequently happens that the interior is hollow, while the sides of the cavity are lined with finely formed crystals of quartz. M. de Verneuil is now engaged in figuring this shell for the Memoirs of the Geological Society of France. Besides those mentioned, our cabinets contain two species of Euomphalus, and one of Murchisonia from the same stratum.

Immediately above and resting on the coralline beds, we have the Shell beds of the Falls. These consist of an assemblage of strata, the different layers of which vary much in their lithological characters. Some are of a light greyish color, compact and sub-crystalline, withstand the action of the atmosphere without much alteration, and answer well for building purposes; whilst others disintegrate when exposed for any length of time to the weather. It is above this point that the *water lime*, used so extensively as a hydraulic cement, occurs.

It is on the Falls that these rocks may be studied to the best advantage, as the strata are here so plentifully charged with organic remains, that it is not easy to find a rock, which on being broken does not yield traces of them. From the Falls they extend in a north-easterly direction, forming the surface rocks for the distance of more than fourteen miles. On the road leading from Jeffersonville to Charlestown, Indiana, there are many fine localities for obtaining organic remains.

The fossils are silicious, while the rocks are soft, and crumble down into a reddish brown dust when exposed to the air, leaving the former exposed on the surface in a good state of preservation.

The lowermost layers of this group contain the following fossils:—

*Pleurorhynchus trigonalis.* (Hall).—A shell, which cannot be distinguished from this species found in the Corniferous limestone of New York, is common on the Falls, and on the Charlestown road, seven miles from Jeffersonville, Indiana. It is calcareous at the former, and silicious at the latter locality. We have occasionally found this shell extending into the coralline beds.

*P. alæformis*—is associated with the above, on the Falls, and also occurs on Lewis's creek, Harrison county, Indiana. In Europe, it is found in the Devonian rocks of Plymouth, Eifel, and Paffrath.

*Atrypa scitula.* (Hall).—We have no hesitation in pronouncing a little species in our cabinets from the Falls, identical with A. scitula from the Corniferous limestone of New York.

*Terebratula reticularis.*—This is the most common and characteristic of all the fossils of the shell beds; it has also the widest vertical range, extending from the base of the coralline beds through the various layers as far up in the series as the slute beds; nor is it confined to these strata, for we even find it in the upper Silurian rocks, which we have mentioned as occurring on Bear-Grass creek. In New York it is found in the Corniferous and Hamilton groups; and in Europe it occurs at St. Petersburg and Gothland, in Silurian strata, while at Plymouth, Eifel, Paffrath, and a number of other places, it is contained in Devonian rocks. It is calcareous on the Falls, but on the Charlestown road it is silicious, and here the spiral appendage is sometimes found beautifully preserved.

*Lucina proavia.* (Goldf).—This species is rare on the Falls, but is more abundant on the Charlestown road. It closely resembles Hall's figure of *Paracyclas elliptica*, in the New York Geological reports, from the Corniferous limestone. Our fossil, however, is double the size of that which is there represented. It seems to hold the same stratigraphical position as the New York species, and is associated with the same fossils.

*Strophomena undulata.* (Vanux).—We have found this shell on Bear-Grass creek, and in Floyd county, Indiana, six miles from New Albany, in strata belonging to the shell beds. In New York it is a Corniferous species. We are not aware of its having been obtained from any other locality in the West.

Several other species of Strophomena characterize our shell strata, one of which resembles *S. radiata* from the Delthyris shaly limestone in New York. If identical, it holds in our rocks, as Mr. Hall observes, a much higher position than in the New York formations.

*Pileopsis tubifer.*—We have in our cabinets specimens of this shell from the Falls, with spines preserved measuring an inch in length. It also occurs on Lewis's creek, in Jefferson county, Indiana.

*Calymene crassimarginata.* (Hall).—The post abdominal portion of this trilobite is very common wherever the shell beds are exposed. The best locality, however, for obtaining good specimens is the Falls. In New York this fossil is from the Corniferous limestone.

*Odontocephalus selenurus.* (Conrad).—This species is associated with the above, but is a rare fossil in our formations. At Schoharie and other localities in New York, where the Corniferous rocks are exposed, it is said to be common. In the absence of a description and perfect specimens, we were for a long time in doubt as to the identity of the New York and western species; but a few weeks since we were so fortunate as to obtain an entire specimen, showing the tooth-like processes on the buckler, precisely as they are seen in the figure in Hall's Report. Our trilobite is much larger than that figured in the New York Reports, the post abdomen of some specimens in our cabinets being nearly four inches in their greatest diameter. We are not aware of its having been obtained from any other locality but the Falls.

*Pterinea cardiiformis?* (Hall).—We are not altogether certain that our Pterinea from the Falls is the same as that described by Mr. Hall under the above name, but it closely resembles the figure in the New York Reports, and holds the same geological position.

Of *Acroculia*, we find here three species which are probably undetermined; one variety we have regarded as identical with the *A. erecta* of Hall.

*Pentremites Verneuili.*—This beautiful fossil, which has been described by Professor Troost, in the Memoirs of the Geological Society of France, is as far as we are informed peculiar to the western States. It is commonly known as "the petrified hickory nut," to which in general appearance it bears some resemblance. It occupies the uppermost stratum visible near Jeffersonville, on the Indiana side of the Falls, and has also been obtained on Bear-Grass creek one mile and a half from Louisville, on Silver creek, at Carr's mills, at Charlestown, Indiana, and in the Cliff Limestone near Columbus, Ohio, in rocks equivalent to our shell beds. We obtained from the same layer, on Bear-Grass, a Pentremite which differs from any with which we are acquainted. It resembles more closely than any other a species given to us by M. de Verneuil from the Carboniferous formations in Yorkshire, England. In the pentremital stratum are to be seen many stems of *Encrinites*, and we have been so fortunate as to find the bodies of four species which are probably new. Two of them belong to the genus *Actinocrinites*; another, related to a different genus, measures five inches in diameter.

The remarkable ganoid fish figured and described by Drs. Norwood and Owen, under the name of *Macropetalichthys rapheidolabis*, from Lewis's creek, Jefferson county, Indiana, in Silliman's Journal, (vol. 1st, p. 367, new series), was broken from a layer which is equivalent to our shell beds. A short time after our attention was called by this publication to the existence of such remains in our formations, we visited the Falls, in company with Dr. Norwood and M. de Verneuil, and were successful in finding a well preserved scutcheon plate of the same species; since which time we have found fragments of other species of fish associated with it, at the same locality, and in the same rocks at points remote from the Falls.

Of *Polyparia* we find several varieties in addition to those which are common to this group and the coralline beds. They are chiefly reticulated corals, among which the *Gorgonia infundibuliformis?* is of most frequent occurrence, as it is also considered the most characteristic. When first split from the rocks it is of a beautiful white, chalky appearance. The best locality for obtaining perfect specimens is at the Falls on the Indiana side, where it may be found in abundance. It corresponds closely to the figure and description given by Goldfuss of this species from the Silurian strata of Europe. If they are identical the American fossil holds a higher position than the European, for it occurs here in strata which are considered as the equivalent of the Devonian rocks of Europe. It is proper to remark, that Dr. Clapp has decided that this Gorgonia is not the *infundibuliformis.*

Immediately above the lower division of the shell beds of which we have been speaking, we reach the upper strata of this group, which consist of a number of layers varying in color from a light blue, to an ashy or greyish tint, and differing much in compactness. One stratum is composed of an aggregation of black rounded pebbles, held together by a kind of scoriaceous looking matter, which crumbles readily on exposure to the air, leaving the surface of the strata in some places covered with these granular bodies. These strata are scarcely less fossiliferous than the lower division of the shell beds, for almost every layer yields objects of interest to the collector. The fossils, however, do not present so great a diversity of species.

Resting on the pentremital stratum we have the water limestone so useful in an economical point of view.

The following are among the most characteristic organic remains belonging to this group:

*Spirifer ostiolatus.* (Schloth).—This shell is one of the most abundant, and characteristic fossils belonging to the water limestone. The best locality is Corn Island, on the Kentucky side of the Falls, where it occurs in both a silicious and calcareous state. The silicified specimens when broken, often exhibit the spiral appendages beautifully agatized, while the space between these and the shell is filled with crystallized carbonate of lime, which is easily removed by dilute muriatic acid, leaving the delicate spires untouched. It is also found silicious on the Charlestown road, seven miles from Jeffersonville, and on Lewis's creek, Jefferson county, Indiana. In the white Limestones of the Red Cedar and Wapsinonox, in Iowa, it is associated with fossils similar to those which accompany it in the shell beds of the Falls. The shell described by Conrad as *Delthyris mucronata*, from the Hamilton group of New York, is undoubtedly the *S. ostiolatus*, for we find that it is associated there with the same organic forms, and the figures of this shell, with its varieties, in the New York Reports, would answer equally well as a description of the western fossil. In Europe it is found in the Devonian rocks of Eifel, Newton, and at other localities.

With this there is another species, which cannot be distinguished from that figured by Mr. Hall as *Delthyris congesta*, also from the Hamilton group, of Seneca lake shore. Again, we find *Terebratula concentrica*, and *T. aspera*, though of unfrequent occurrence, both on the Falls and in Floyd county, Indiana. The former characterizes the Hamilton group of New York, and the Devonian strata of Eifel, Russia, Spain, and Belgium; while the latter is found at Gothland and Eifel. This limestone also yields four undetermined species of Terebratulæ.

Of the new genus *Chonetes*, we find one species exceedingly abundant—the *C. nana*, figured and described in the "Gé-

ologie de la Russie," (vol. 2d, p. 245, pl. xx, fig. 12). This species is exclusively Devonian, and in Russia occurs on the shores of the Don, near Voroneje. On the Falls it is mostly silicious, and in some of the disintegrations on Corn Island it may be obtained with its delicate spines well preserved.

Of *Pteropoda* we have two species. One of these is the *Conularia quadrisulcata* of Miller. This also occurs at Button-mould Knob, seven miles south of Louisville, in rocks which are considered as belonging to the Carboniferous system. This is an interesting fossil, as it is one among the few found in Kentucky common to two formations. In New York it occurs only in the Niagara group. An undetermined species occurs in the uppermost limestone of the Falls.

The *Calymene macrophthalma*, (*C. bufo. Green*), is quite characteristic of these upper shell beds, and is to be met with wherever these strata are exposed. In New York, it occurs in the Hamilton group; in Iowa, in the white Limestone of Red Cedar and Wapsinonox; in Indiana, it is found near Charlestown; its foreign localities are Yorkshire and Eifel. Some of our specimens are calcareous, but many are silicious.

Our cabinets also contain several corals from this stratum, among which we may mention *Cystiphyllum vesiculosum*, and *Favosites basaltica* as most abundant.

On Corn Island the water Limestone is covered by a silicious crust, which is not more than two inches in thickness. In this crust we find a small *Orthoceratite*, two, and sometimes three inches in length, with very thin septa. We have not been able to detect the position of the syphon. It is always silicious. The *Loxonema Hennahiana*, a beautiful spiral shell is very abundant at this place, and, so far as we know, this is the only locality in the western States where it has been obtained. It is figured by Phillips in his *Palaeozoic Fossils*, pl. 38, fig. 104. Its foreign localities are South Devon and Eifel, where it occurs in Devonian rocks. A small *Terebratula* and a *Turbo*, both of undetermined species, are also found here.

Immediately above, and resting on this stratum, we have a layer of a granular structure, which contains numerous species of Encrinites, with a few corals and shells. The heads, or bodies of the former are also, now and then, obtained, and our cabinets are enriched by several species from this locality. One of these belongs to the genus *Actinocrinites*, and another is perhaps a *Cyathocrinite;* whilst a third, which Professor Troost is figuring and describing for the Memoirs of the Geological Society of France, seems to be distinct from any genus yet published.

The *Slate beds* are superimposed upon this Encrinital stratum, and are best seen at New Albany, Indiana. Dr. Clapp by boring has ascertained their thickness to be one hundred and four feet at that point. In the excavations for the Louisville and Portland Canal, these beds have been cut through, their thickness on this side of the river being much less than at New Albany. They form the surface rocks for the distance of seven miles south by west from Louisville, where they are overlapped by the fine-grained sandstone of the Knobs presently to be described. The black slate here, as well as at other places where these rocks occur, are sparingly fossiliferous, only a few organic forms occurring in them, and these being confined to one or two of the lowermost layers of the mass. When newly fractured the slate is of a jet black color, owing, probably, to the great amount of bituminous matter which it contains, but after long exposure to the air it assumes a much lighter shade. Iron pyrites freely disseminated throughout the mass, either in the form of thin laminæ, or in small nodular masses, imparts to the water of the Slate district the taste of sulphate of iron. The only fossils which we have found in this formation are a small *Lingula*, and an *Orbicula;* the former, from the figure in the New York Geological Reports, is probably *L. concentrica*, (Vanuxem) from the Genessee slate; the latter, *O. Lodensis* (Vanux.) from the same slate.

Near White's creek springs, in Tennessee, on Paradise

Ridge, according to a barometrical measurement måde last summer by Drs. Owen and Norwood, the black slate is 51·8 feet thick. Near the base of the hill these gentlemen found a small Lingula, which bears a strong resemblance to *L. spatula*, of the Genessee slate.

It will appear from the preceding observations, and from an examination of the table of fossils found in the vicinity of Louisville identical with European species, appended to this article, that the organic remains of which we have spoken as occurring in the strata on Bear-Grass creek, and on the Falls beneath the coralline beds, are decidedly of a lower Palæozoic type. Of twelve determined species therein enumerated as characterizing these rocks, seven forms are common to the Silurian and Devonian systems of Europe; viz: *Terebratula Wilsoni*, *T. reticularis*, *Spirifer trapezoidalis*, *Leptena depressa*, *Catenipora escharoides*, *Stromatopora polymorpha*, and *Cyathophyllum dianthus;* the remaining five species, *Spirifer lynx*, *Orthis testudinaria*, *Leptena sericea*, *Pentamerus oblongus*, and *Calymene Blumenbachii* are peculiar to the Silurian rocks. M. de Verneuil spent several days, last summer, investigating the fossils of this vicinity, and his examination enabled him to identify a number of forms which in Europe occur only in Devonian rocks; from which he was led to consider the upper beds of the Falls as belonging to that system, and to conclude that the line of separation between this system and the Silurian, is about the base of the upper coralline beds.

An examination of the tables will show twenty species from the upper beds of the Falls identical with forms occurring in the Devonian system of Europe. Of these ten are common to it and the Silurian system; viz: *Terebratula reticularis*, *Spirifer ostiolatus*, *Calymene bufo*, *Favosites polymorpha*, *F. basaltica*, *F. fibrosa*, *F. Gothlandica*, *F. spongites*, *Aulapora serpens*, and *A. tubiformis;* the remaining ten, viz:—*Spirifer cultrijugatus*, *Pleurorhynchus alaeformis*, *Productus subaculeatus*, *Chonetes nana*, *Loxonema Hennahiana*, *Pileopsis*

*tubifer*, *Lucina proavia*, *Venulites concentricus*, *Cystiphyllum vesiculosum*, and *Reptepora infundibulum?* are forms of organisms, purely Devonian.

Such is the result of the comparison we have made; from which it appears, that a decided analogy exists between the fossils of the upper beds of the Falls, and those of the Devonian strata of Europe, and that there are sufficient grounds for referring them to that geological epoch.

We may next compare the species contained in the western Silurian and Devonian strata with their representatives in New York.

The first table at the end of this article shows thirty-one species occurring here, which may be considered as identical with New York forms.

Of these it will be seen that the Clinton group is represented by two species, the Niagara group by six, the Onondaga limestone by five, the Corniferous limestone by nine, the Hamilton group by eight, and the Genessee slate by two; while from the above comparison, the following formations occurring in New York, above the Clinton group, appear to have no representatives in the western States; namely: the Onondaga slate and water-lime groups, the Pentamerus limestone, the Delthyris shaly limestone, Oriskany sandstone, Marcellus shale, Tully limestone, and the Portage and Chemung groups. The fossils in our strata, of which we have spoken, correspond in the order of their superposition precisely with those of New York.

Dr. Clapp has determined the thickness of all the strata of the Falls but the lowest, and we are indebted to him for the following summary, exhibiting at one view their succession and general depth:—

STRATIFICATION OF THE FALLS OF THE OHIO.

| | |
|---|---|
| UPPER LIMESTONE. - - | Sub-crystalline limestone 8 feet.<br>Water limestone 12 feet=20. |
| SHELL LIMESTONE. - - | Sub-crystalline limestone with many characteristic shells and trilobites, and a few corals - - - 16 feet. |
| CORALLINE LIMESTONE. | *Upper coralline* to catenipora, composed mostly of corals, and destitute of shells, - - - - - - 20 feet.<br>*Lower coralline*, corals mostly different from those above, and very few shells—the upper part alone visible on the Falls, - - 20 feet=40+. |

Seven miles south of Louisville are seen the hills known as the "*Button-mould Knobs*," in which the "Encrinital limestone" of Professor Troost occurs. These hills are composed, for the most part, of a fine-grained Sandstone, and the limestone containing the crinoidal remains appears to be a local deposit not to be seen in most of the knobs. The uppermost layers of this sandstone on many of the hills abound in a hydrated oxide of iron, commonly called "kidney ore," from which iron of a superior quality is manufactured at furnaces near Shepherdsville, on Salt river. This formation is poor in organic remains. We have found the *Orthis crenistria* and the *Spirifer cuspidatus* at a single locality; the *Conularia quadrisulcata* and a *Phillipsia* have been found in nodules of iron ore on its surface, and besides these we have not been able to detect any fossils in the fine-grained sandstone. In Europe, *O. crenistria* occurs in Devonian rocks, and at one time we were inclined to believe that our fine-grained sandstone belonged to the same system; but subsequent researches have satisfied us that it must be referred to a later geological era. *S. cuspidatus*, in Eifel and South Devon, is found in the Devonian formations, whilst in Ireland, as with us, it occurs in rocks belonging to the Carboniferous system.

Intercalated in the Sandstone, on a few of the knobs, is found the encrinital limestone, remarkable for the number and beauty of its fossils. Two knobs have been discovered in Jefferson, and one in Bullitt county, where this limestone is seen, and among the equivalent hills in Allen county, Kentucky, and at White's creek springs, in Tennessee, this peculiar formation also appears. This limestone which is little else than a mass of the remains of Encrinites is wanting in all the knobs near the river on the Indiana side, though we believe we have recognized it in some of the hills a few miles south of Salem, where, as at other points, it occurs among the layers of the Sandstone. Its depth below the surface of this rock varies from fifteen to a hundred feet, owing probably to the extent to which the Sandstone has undergone abrasion.

The elegant species of *Actinocrinite, plate* figure 5, *b*, is from this formation, and was obtained from the knob seven miles south of Louisville. Dr. Troost has found the same encrinite in Tennessee, at White's creek springs, and is figuring and describing it for the Memoirs of the Geological Society of France. The proboscis of this specimen is an inch and a half in length, and we have also found this appendage, belonging probably to the same species, of various shapes, sometimes bifurcated, and again assuming an irregularly branched appearance.

Besides this actinocrinite we have found here eight or ten undescribed species of crinoideans, and the plates of probably as many more, three of which we have been enabled to restore so that their specific characters can easily be made out. The plates, costal and pelvic, of one species accord very fully with the figure and description given by Goldfuss of *Cyathocrinus geometricus*. We have also found here the plates of a new species of *Pentremite;* and in the corresponding formation of Allen county, Kentucky, we obtained a beautiful species—the *P. granulatus* (Troost).

The localities which afford these interesting fossil forms also abound in Shells, among the most characteristic of which are the following species:

*Orthis Michelini.*—This shell occurs most abundantly in the superior layers of the bed, where it may be obtained in countless numbers, especially at the knob in Bullitt county. It is often seen crushed and contorted into various shapes. Its geographical range is very great, for we find it described in the *Géologie de la Russie* as occurring in the Carboniferous rocks of the Oural mountains, in Russia; and it is also found in Belgium and in England.

*Spirifer undulatus.*—This shell is collected with the one just described, but is by no means so abundant, good specimens being rarely found.

*Spirifer striatus.*—This shell, which has also a wide geographical range, is three or four times as large as its European congener. In Europe it is considered one of the most characteristic of the fossils found in the carboniferous rocks.

*Terebratula Roissyi.*—This shell is somewhat common at this locality. One specimen in our possession exhibits in great perfection the spiral appendage. This fossil also belongs to the carboniferous system of Europe.

*Productus punctatus.*—This highly characteristic shell occurs abundantly with those above enumerated, and is often accompanied by

*P. semireticularis.*—These fossils, the authors of the Geology of Russia regard as more characteristic than any others of the carboniferous rocks of Europe. Their position is below that of *Orthis crenistria* and *Spirifer cuspidatus*, found in the sandstone of the knobs, and hence we conclude that that formation belongs to the carboniferous system.

In addition to these we find here many other species of shells, among which we may mention *Chonetes Dalmaniana* with the characteristic spines well preserved; *Conularia quadrisulcata*, and *Buccinum acutum;* besides a number not yet determined belonging to the genera *Pileopsis*, *Leptena*, *&c.*

Of *Crustaceæ* we find here two species, one of which resembles *Phillipsia Ouralica*, from the carboniferous rocks of the Oural mountains, figured and described in *Géologie de la Russie*, page 378, figure 16, *a*, *b*. The other belongs to the genus *Griffithides*, but its species has not been determined. Both fossils are rare.

Few *Polyparia* are found in this formation, and these belong principally to the genera *Cyathophyllum*, *Cyathoxonia*, and *Aulopora*. With the exception of a single species—*Cyathoxonia cornu*,—none have been determined.

There can be no doubt in regard to the character of this formation, for of the twelve species enumerated in the table as identical with European forms, all except one—the *Cyathocrinus geometricus*—are there peculiar to the carboniferous rocks.

Near the mouth of Salt river, on the Nashville road, the sandstone of the knobs disappears under the Carboniferous limestone. This river runs much of its way among hills composed of fine-grained sandstone, and its falls at Shepherdsville, are formed of strata which are identical with those at the Falls of the Ohio. For several miles after crossing it at that place, in a direction south, these strata are seen occasionally on the surface, and we have remarked in them the same profusion of encrinital remains found among the upper strata of some of the rocks in the Ohio, at Louisville, and have procured from them portions of a Fish, perhaps appertaining to the species plates of which we have found at the Falls. On the sides of the hills in this neighborhood, the nodular iron ore of which we have spoken occurs. Some specimens of this ore abound in the stems of encrinites, owing to which it has received, from the workmen, the name of "chocolate ore;" in others we have found the Phillipsia, which we are disposed to regard as *P. Ouralica*.

Ascending the turnpike south of Salt river, about midway the cliffs, the pentremital layers of the mountain limestone come in view, and near the summit to the east of the road, we have found an undescribed encrinite, of the genus *Actinocrinites.* From this point to Elizabethtown, and ten miles west of that place, in the direction of the Grayson Springs, the road lies over the compact, cavernous limestone marked almost everywhere by the presence of the Barrens. Owing to this cavernous quality of the Carboniferous limestone, but few streams of water are seen in districts of country where it is the prevailing rock, while caves, subterranean streams, and sink-holes every where abound. The rivulets which descend from the hills of coarse-grained sandstone, next to be mentioned, sink and disappear as soon as they reach this limestone. This is the formation which presents us with the Mammoth Cave, and numerous other caverns of hardly inferior magnitude exist in its uppermost strata. These strata are remarkable for the regularity with which they afford the *Pentremites globosa* and *P. florialis.* We have collected specimens of these in the Mammoth Cave, and from the same interesting locality have an encrinite of the genus *Platycrinus*, which we took from the ceiling of the Fairy Grotto. Among the fossils found on the surface of this rock, at points near Elizabethtown, and in the vicinity of the Cave, we may mention the *Lithostrotion floriforme*, *Syringopora ramulosa*, and a number of undetermined species, belonging to the genera *Euomphalus*, *Productus*, *Bellerophon*, and *Terebratula*. They are generally found silicified, especially the corals, the rocks in which they were originally imbedded having worn away, and left them exposed on the surface.

On the road to Litchfield, about ten miles west of Bowling-green, the coarse-grained Sandstone, the first of the coal series, makes its appearance in an abrupt hill, and from this point to the Grayson Springs it is the surface rock, except where it has been eroded by streams of water down to the mountain limestone. This is the case at the Springs, and it was in the superior strata of the limestone at this place, that we met with the richest deposit of *Crinoidea* which we have anywhere yet found. Prof. Cobb had visited the Springs before us, and the beautiful specimens which he brought away, one of which Drs. Owen and Norwood have figured (see their Researches among the Protozoic and Carboniferous Rocks of Kentucky) induced us to make a thorough exploration of the locality. The spot at which these fossils were preserved in such perfection, is within three hundred yards of the mineral springs, and is so limited in extent, that we left it with the impression that we had exhausted it of its treasures. In this narrow space, not exceeding forty feet square, we found seven or eight species of Encrinites, belonging to nearly as many genera. The rock in which they were imbedded is the superior layer of the limestone, immediately under the coal series. A spring of water that issues after copious rains had washed out and exposed some of the finest specimens; many were picked up on the top of the ground, and others were raised a few inches below the surface. Beyond these narrow limits we searched the rocks in vain for the fossils which we had found there in such profusion, and the fact appears to us worthy of note, as illustrating the habits of this extinct race of beings. As at this place, their remains, we believe, have generally been found in circumscribed deposits, showing a gregarious tendency in the animals. The lily encrinite of Europe seems to be confined to a single locality. We have mentioned that the fine *Actinocrinite* figured in the plate, occurs at but a single locality in Kentucky, and the *Caryocrinus ornatus* appears in our neighborhood in a single stratum. The *Cyathocrinus ornatissimus*, of Hall, appears to be quite as limited in its distribution.

How many of the Encrinites discovered in Grayson are new, we have not been able to satisfy ourselves in the absence of the latest works on the Crinoidea, but, most of them we take to be undescribed. Several belong to the genus *Cyathocrinus*, and some do not come within the definition of any of the genera known to us. One, *plate* figure 1, we name, provisionally, *Cyathocrinus florialis*, presenting as it does, with its long arched stem, a striking resemblance to a flower; and for the one resembling the human hand with the fingers compressed and extended, figure 2, we propose the name *Cyathocrinus maniformis.* The very remarkable form, figure 3, is unlike any drawing we have seen, and will probably originate a new genus. The costal plates very far exceed in number those of any of the genera of Miller. We found but few specimens of this interesting encrinite. The individual figured, is the more remarkable from the presence of the shell at the entrance into its stomach. The animal would appear to have been in the act of gorging an *Acroculia* at the moment when it perished, and we should be more inclined to favor this conjecture if the prey were less disproportioned to the organs of the encrinite. The carnivorous habits of the crinoideans, we believe, have been clearly made out.

These encrinites, with several other species, occur at the locality near the Springs. The last one, *plate*, figure 4, a, b, c, d, was found two miles north of Litchfield, and six miles from the Springs. Of this we collected about a hundred specimens, on a surface but a few yards in extent, and although we examined the country many miles around we found no traces of this fossilized form elsewhere.

Our attention was chiefly directed during our excursion to the discovery of Encrinites, but collocated with these fossils we found *Archimides*, *Pentremites florialis*, *P. globosa*, *Terebratula lamellosa*, *Spirifer striatus*, and *Productus punctatus*, together with many undetermined species of brachiopods.

Immediately above the limestone filled with these remains, appears the Sandstone in which coal is deposited. Coal is found in various parts of Grayson county, and some beds afford it of a very good quality, though generally they are too thin to be worked with profit.

With the limestone, nearly all traces of animal life disappear, only a stray fossil here and there being found as high up as the sandstone. In the lowermost beds of this rock, resting upon the limestone, we detected a few shells belonging to the genus *Orthis;* and in a stratum of yellow stone, which seemed to be composed of the two formations, we procured *Terebratulæ*, the teeth of one or two species of

*Fish*, together with fine specimens of a *Productus* with its long spines preserved. In strata about three hundred feet above the point where these fossils cease, coal is deposited.

Mineral Springs.—The region of which we have been speaking possesses several mineral springs, of which perhaps some notice would be expected in a memoir like the present. These Springs vary in their constitution as they are found issuing from different mineral strata, though with few exceptions they agree in containing sulphuretted hydrogen as one of their ingredients. Those found in the Cliff limestone,—the strata of the Falls,—are characterized by the large amount of *chloride of sodium* suspended in their waters; those of the Slate formation are impregnated with *iron;* while in the Grayson Springs, which occur in the Carboniferous limestone, the characteristic ingredient is *magnesia.* The sulphuret of iron, which is detected everywhere in the black Slate, accounts for the chalybeate qualities of the water percolating its beds. This sulphuret soon becomes a soluble sulphate, under the influence of moisture and oxygen, and is in a condition then to be washed out by the rains, and appear as a chalybeate spring. The sulphuretted hydrogen, which is so uniform a constituent of all these springs, no doubt owes its origin to the same sulphuret, that portion of its sulphur which is in excess combining with the hydrogen of the decomposed water to form the gas. Chloride of sodium may have been left in basins in the rocks when the sea, which there can be no doubt at one time covered them, receded into new channels; or it is possible that it exists there in the form of rock salt, in which state it is known to occur in many countries. Either hypothesis explains the presence of this salt in so many mineral waters. Magnesia enters into the composition of many rocks, and it is easy to perceive how the carbonic acid carried down by the rains, and the sulphuric acid developed from the iron pyrites by the process just alluded to, would render it soluble, and bring it to the surface in the shape of a carbonate and sulphate.

*Paroquet Springs.*—In a notice of these Springs in this Journal (vol. vii, p. 143, new series) it was stated that they occur in the Blue limestone. They occur not far from the point where the two formations meet, but, as has been mentioned above, among the beds of the Cliff limestone. They belong to a series of springs to which Salt river is indebted for its name. All the salt consumed in this region of Kentucky, for many years, was made from wells the remains of which, with the works for raising and transporting the salt-water, are still to be seen near this river in the vicinity of Shepherdsville. The discovery of stronger brine on the Kenawha and other places, led to such a reduction in the price of this commodity that its manufacture at these works ceased to be porfitable. The Paroquet Springs differ from the wells in the neighborhood in being charged with sulphuretted hydrogen, but like the other fountains they hold in solution much table salt. Their other constituents are chloride of calcium, chloride of magnesium, sulphates of soda, magnesia and lime, and carbonates of lime and magnesia. The water is cold, and in its action purgative and alterative, adapted to cases of chylopoietic derangement, and, generally, to affections growing out of sedentary habits.

Within a few hundered yards of the main spring is another fountain, in which sulphate of magnesia is the predominating ingredient, and common salt and sulphuretted hydrogen are not found.

In the same formation, a short distance from Charlestown, Indiana, a feeble stream of salt water issues, which in early times was the resort of buffalo and deer. It is destitute of sulphuretted hydrogen, but in other respects resembles the Paroquet Springs.

The Chalybeate Springs and wells of the black slate formation have not attracted public attention; nevertheless their waters might be used with advantage in anemic and other diseases demanding a tonic treatment.

*Grayson Springs.*—These Springs are within four miles of Litchfield, the county seat of Grayson, and twenty miles north of the Mammoth Cave. The number of fountains is very great, though only slight differences have been detected in the character of their mineral ingredients. They are all highly charged with sulphuretted hydrogen, and contain in addition, carbonic acid, carbonate of magnesia, carbonate of lime, sulphate of magnesia, and sulphate of lime. A trace of iron has been detected in several of the springs. Their temperature is as low as that of the best common springs in the neighborhood, about 58° Fah., and their taste sweetish and not unpleasant. They have acquired much reputation for their curative powers in a variety of chronic disorders. The water acts gently upon the bowels, promotes the secretion of the kidneys and the skin, and improves the appetite and digestion. Another spring has been discovered a few miles distant from these, in the same formation, and possessing all their qualities.

In a general survey of the several formations passed in review in this memoir, the following are some of the facts which will strike the observer. He will remark, that wherever the Cliff limestone constitutes the superficial rock, the soil is rich and favorable to agriculture. Next to the regions of Kentucky in which the Blue limestone prevails, the best soil is found where the rocks of the Falls are the prevailing formation. After this comes the Carboniferous limestone of the Barrens, where the soil, although still good, is inferior to that which reposes upon the series just mentioned, with the additional disadvantage that, owing to the cavernous quality of the rocks, it is not well watered. The soil over the Black slate is adapted to the culture of small grain crops, and especially to grass, but does not produce corn or hemp as well as the limestone districts, owing to its tenacious consistency, which subjects it to the formation of ponds and marshes, and renders it generally too retentive of moisture. The regions of the Sandstones are poor; they embrace the 'Knobs' and 'wildernesses' of Kentucky.

The relations of these various geological groups to health and disease, are both interesting and important. Malarious affections are known to be dependent upon the joint agency of heat and moisture, and their prevalence is generally graduated by the extent to which these influences are combined. With this principle before him, the observer would be prepared to find fevers more prevalent over our Slate districts than in the others; and if he met with an exception to the rule, it would be in the Barrens, where, owing to the predominance of a tough red clay in the soil, ponds have accumulated in greater numbers. Along the streams in the Blue and Cliff limestone districts fevers prevail, but over the entire surface of the Slate, they are expected, in wet seasons especially, as regular summer and autumnal occurrences. The early history of Louisville with regard to salubrity, is well known; it was visited, in different years, by epidemics rivaling in malignity the yellow fever of New Orleans.—Built upon the margin of the Slate, it was at that time surrounded by ponds of great extent. These have been drained; the country around has been cleared and brought under cultivation, and while the "Pond Settlement" continues as unhealthy as formerly, Louisville, in latter years, has come to be a healthy city.

The Mammoth Cave and the Falls of the Ohio, will occur to every observer as the most remarkable objects in the geology of the portion of Kentucky under consideration. The caves, which on account of their size have attracted attention in this country, are all found in the Carboniferous limestone, and it is interesting, as we believe we are authorized to remark, that from Palestine to the Mammoth Cave, those of the greatest magnitude occur in the same formation. The common theory of these excavations is, that they have been wrought out slowly in the rock, by the united action of carbonic acid and water, and it cannot be doubted that these agents have shared largely in the process; but an attentive consideration of the phenomena has led us to suppose that nitric acid also has contributed to it. Nitrate of lime is everywhere met with on the floors of these caverns, except under circumstances where water has been admitted to wash it away, showing that a portion of their solid walls has assumed that state. How much of the erosion is due to the latter acid it would not be easy to show, more especially as, from the soluble character of the nitrate of lime, but little of that salt could remain where water had access to the cave; but all the facts considered, is it not evident that nitric acid has had its share in these excavations?

The Falls of the Ohio constitute a *coral reef* unequaled, perhaps, by any bed of ancient corals known in the world. The strata exposed for miles are almost wholly made up of these fossils, many of them silicious, and all of a compact, resisting character. In this quality of the rocks, owing to which they have yielded more slowly than the strata above to the abrading influence of the river, we have, doubtless, one of the causes of the Falls. Some of the rocks, more fully charged with corals than the rest, have defied the action of the water, while in other places, where less fossiliferous, they have gradually given way and are worn into deep channels. Other causes have contributed to produce these rapids in the Ohio. The dip of the strata, though not uniform, is generally in the direction here taken by the stream, and is also greater than at points above. But, added to this, the formation succeeding to the Cliff limestone, at the Falls, is of a far more friable character, and yields rapidly to the force of the river. The limestone continuing, we should have been presented with extensive rapids, but the fall would have been slight. As it is, the water has worn away the Slate, and follows the dip of the limestone, creating a fall of more than twenty-two feet in a distance of two miles.

The Falls of Niagara occur in formations analogous to those at the Falls of the Ohio, but the relative position of the rocks is reversed. In Niagara the destructible clay slate is underneath the limestone; in the Ohio it is above, and

6

this creates the difference between the Falls of the two rivers. If our clay slate had been the underlying rock, then our Falls, instead of rapids, would have been a cataract.

The denuding effects of water are exhibited on an imposing scale at the Falls of the Ohio. According to an Indian tradition, the river at one time flowed on what is now the Kentucky side of Corn Island, which then was connected with the mainland on the north; and in the recollection of many of the present inhabitants of Louisville, that island extended up as high as third street, opposite the mouth of Bear-Grass. The river now, except at high stages, flows almost exclusively on the Indiana shore, and the island reaches only to 11th street, two-thirds of a mile in length having been washed away in less than the third of a century. These changes have taken place in the memory of man, but there is conclusive evidence in the strata around that far greater mutations occurred in ages more remote. At one period, the clay slate strata, a hundred feet in thickness, reposed upon the limestone at the Falls. These have all disappeared, and, borne towards the sea, have taken part in other formations. The hills of Silver creek and the Button-mould Knobs were once united; they are now seven miles asunder, all the intervening mass of rocks, more than a hundred yards in depth, having been carried away by running water. And if we conclude, as there are strong grounds for believing, that the mountain limestone of Kentucky was once connected with its equivalent in Indiana, and that the great coal fields of Illinois, Ohio, and Pennsylvania are but parts of what at one period was a continuous field, then we have many thousand feet of solid strata, several hundred miles in extent, which have yielded to this slow process of denudation.

A TABLE OF FOSSILS FOUND IN THE VICINITY OF LOUISVILLE IDENTICAL WITH NEW YORK SPECIES.

| No. of Species. | GENERA AND SPECIES. | Authors and references. | Silurian System. | Devonian System. | FORMATIONS IN NEW YORK. Clinton Group. | Niagara Group. | Onondaga Limestone. | Corniferous Limestone. | Hamilton Group. | Genesee Slate. |
|---|---|---|---|---|---|---|---|---|---|---|
| 1 | Catenipora escharoides | Lam. | * | | | * | | | | |
| 2 | Favosites Gothlandica | Lam. | | * | | | * | | | |
| 3 | Astrea rugosa? | Hall | | * | | | * | | | |
| 4 | Cyathophyllum dianthus? | Goldf. | | * | | | * | | | |
| 5 | Syringapora? | | | * | | | * | | | |
| 6 | Pentamerus oblongus | Sil. res. | * | * | * | | | | | |
| 7 | Pterinea cardiiformis | Hall | | * | | | | * | | |
| 8 | Terebratula reticularis | Brown | * | | * | * | | | | |
| 9 | " " scitula | Hall | | * | | | | * | | |
| 10 | " " concentrica | Von Buch. | * | * | | | | | * | |
| 11 | " " spinosa? | Hall | | * | | | | | * | |
| 12 | Delthyris mucronata | Conrad | | * | | | | | * | |
| 13 | " zig zag? | Hall | | * | | | | | * | |
| 14 | " congesta | Hall | | * | | | | | * | |
| 15 | " undulatus* | Schloth | | | | | * | | | |
| 16 | Strophomena depressa | Sow. | * | | | * | | | * | |
| 17 | " inequistria? | Hall | | * | | | | | * | |
| 18 | " undulatus | Vanux | | * | | | | * | | |
| 19 | Pleurorhynchus trigonalis | Hall | | * | | | | * | | |
| 20 | Paracyclas elliptica | Hall | | * | | | | * | | |
| 21 | Euomphalus? rotundus | Hall | | * | | | | * | | |
| 22 | Orbicula lodensis | Vanux | | * | | | | | | * |
| 23 | Lingula concentrica | Vanux | | * | | | | | | * |
| 24 | Calymene bufo | Green | | * | | | | * | | |
| 25 | " crassimarginata | Hall | | * | | | | * | | |
| 26 | " Blumenbachii | | * | | | * | | | | |
| 27 | Odontocephalus selenurus | Conrad | | * | | | | | | |
| 28 | Caryocrinus ornatus | Say | * | | | * | | | | |
| 29 | Hypanthocrinites cœlatus | | * | | | * | | | | |
| 30 | Acroculia erecta? | Hall | | * | | | | * | | |
| 31 | Turbo lineatus | Hall | | * | | | | | * | |

* The true Spirifer undulatus occurs in this county in the carboniferous limestone of the Knobs.

### A TABLE OF FOSSILS FOUND IN THE VICINITY OF LOUISVILLE IDENTICAL WITH EUROPEAN SPECIES.

| Number of Species. | GENERA AND SPECIES. | AUTHORS AND REFERENCES. | FORMATIONS IN EUROPE. | | | LOCALITIES. | PRINCIPAL LOCALITIES OF EUROPE. |
|---|---|---|---|---|---|---|---|
| | | | Silurian. | Devon. | Carbonif | | |
| | BRACHIOPODA. | | | | | | |
| 1 | Terebratula Wilsoni..... | Min. Conch., p. 118, f. 3 V. Buch. Foss. Rhen. Prov. | * | * | ........ | Jefferson co, Ky, Perry co, Tenn., Lockport, N.Y. | St. Petersburg, Russia, Norway, England. |
| 2 | " aspera...... | Schloth—Geol. Rus. p. 93, pl. x, fig. 12, Hall's report. | ........ | * | ........ | Falls of Ohio. | Gothland, Eifel. |
| 3 | " concentrica.. | Von Buch—Russ. Geol. vol. 2, pl. viii, fig. 10 and 11. | ........ | * | ........ | Falls of Ohio, Floyd co., Ia., New York. | Russia, Spain, Eifel, Belgium. |
| 4 | " Roissyi..... | Leveille—Geol. Russ., vol. 2, p. 55, pl. ix, fig. 2. | ........ | ........ | * | Button-m'ld Knob, Grayson co. Ky., Floyd co, Ia. | Belgium. |
| 5 | " reticularis... | Sin.—I. prisca Schloth., Russ. Geol., p. 90, pl. x, fig. 12. | * | * | ........ | Falls of Ohio, Bear-Grass creek, near Louisville. | Russia, Turkey, Spain Eifel. |
| 6 | Spirifer ostiolatus........ | Schloth, pl. 17, f. 3, Phil. Palæozoic Fossils p. 30, f. 132. | * | * | ........ | Falls of Ohio, Clark co., Indiana. | Russia, Eifel. |
| 7 | " cultrijugatus..... | C. Roemer. | ........ | * | ........ | Falls of Ohio. | Eifel. |
| 8 | " striatus......... | Sow.—Min. Conch., vol. 3, p. 125, pl. 270, Russ. Geol., p. 16, pl. 6. | ........ | ........ | * | Button-mould Knob, Allen co., Ky. | |
| 9 | " trapezoidalis .... | Sow.—Sil. Syst., pl. 5. fig. 14. | * | * | ........ | Bear-Grass, Jefferson co. | Russia, Belgium, England, Spain. |
| 10 | " undulatus....... | Sow.—Min. Conch., Von Buch. | ........ | ........ | * | Button-mould Knob. | |
| 11 | " cuspidatus ...... | Sow.—Phil. Pal. Foss., p. 72, pl. 29, fig. 124. | * | * | ........ | Floyd co., Ia. | Russia. Russia. |
| 12 | " lynx ........... | Eichw.—Russ. Geol., p. 126. pl. iii, fig. 3. | ........ | ........ | ........ | Jefferson and Bullitt cos., Kentucky. | Russia. |
| 13 | Orthis Michelini......... | Leveille—vol. 1, p. 131, Russ. Geol., vol. 2, p. 185, pl. 13, f. 1. | ........ | ........ | * | Button-mould Knob, Bullitt co., Ky. | Belgium, Yorkshire. |
| 14 | " testudinaria....... | Dalm., vol. 1, p. 408. | * | ........ | * | Jefferson county, Ky. | Russia. |
| 15 | " crenistria......... | Phil., vol, 1, p. 42. | ........ | * | ........ | Floyd county Ia. | Yorkshire, Russia. |
| 16 | Leptena depressa........ | Sow.—Min. Conch., Von Buch. | * | * | ........ | Bullitt county, Ky. | Russia, Eng., Eifel. |
| 17 | " sericea......... | Sow.—Russ. Geol., vol. 2, p. 277, pl. 15, fig. 1. | ........ | ........ | ........ | Bullitt county, Ky. | Russia, Gothland, Eifel. |
| 18 | Productus punctatus..... | Martini—Russ. Geol., vol. 2. p. 276, pl. 18, f. 3. | ........ | ........ | * | Button-mould Knob. | Russia, Belgium, England. |

| | | | | | | | |
|---|---|---|---|---|---|---|---|
| 19 | Productus semireticulatus. | Martini—Russ. Geol., vol. 2, p. 262, pl. 16, fig. 1. | ........ | ........ | * | Button-mould Knob. | Russia, Belgium, England. |
| 20 | " subaculeatus .. | Murch. Sil. Syst., Russ., Geol. vol. 2, p. 282, pl. 16, fig. 9. | ........ | * | ........ | Clarke county, Indiana. | Belgium, France, Eifel, Russia. |
| 21 | Chonetes nana .......... | Geol. Russ., vol. 2, p. 245, pl. 15, fig. 12. | ........ | * | ........ | Falls of Ohio, Bullitt co., Ky. | |
| 22 | " Dalmaniana.... | De Koninck fossils de Belgique. | ........ | ........ | * | Button-mould Knob, Bullitt co., Ky. | Belgium. |
| 23 | Pentamerus oblongus .... | Sil. Syst., vol. 2, pl. 19, fig. 10. | * | ........ | ........ | Bear-Grass creek. | England. |
| | GASTEROPODA. | | | | | | |
| 24 | Buccinum acutum....... | Sow.—Russ. Geol., vol. 1, p. 75, 440. | ........ | ........ | * | Button-mould Knob. | Yorkshire, Russia. |
| 25 | Loxonema Hennahiana .. | Phil. Pal. foss., pl. 38, fig. 184. | ........ | * | ........ | Falls of Ohio. | S. Devon, Plymouth. |
| 26 | Pileopsis tubifer......... | | ........ | * | ........ | Falls of Ohio. | |
| | ACEPHALES. | | | | | | |
| 27 | Lucina proavia . ......... | Goldf. p. 146, fig. 6. | ........ | * | ........ | Falls of O., Clarke co., Ia. | Eifel. |
| 28 | Venulites concentricus... | F. C. Roemer. | ........ | * | ........ | Clarke county, Indiana. | Eifel. |
| 29 | Pleurorhynchus alæformis | Goldf., 146, fig. 6. | ........ | * | ........ | Falls of Ohio. | Eifel, Yorkshire. |
| | PTEROPODA. | | | | | | |
| 30 | Conularia quadrisulcata.. | Miller—Sil. Syst., p. 102. | ........ | ........ | * | Falls of Ohio, Button-mould Knob. | Brook Dale Coal. |
| | CRUSTACEA. | | | | | | |
| 31 | Calymene bufo.......... | Green Monag., Sil. Syst., pl. 14, fig. 2. | * | * | ........ | Falls of Ohio, Clarke co., Indiana, New York. | Eifel, Yorkshire. |
| 32 | " Blumenbachii.. | Brong., vol. 1, p. 401, Russ. Geol., vol. 1, p. 401. | * | ........ | ........ | Westport, Ky., Jefferson co., Ky, Bear-Grass. | Gothland, Russia. |
| 33 | Phillipsia Ouralica?...... | Russ. Geol., vol. 2, p. 378, pl. 27, fig. 16. | ........ | ........ | * | Button-mould Knob, Bullitt co., Ky. | Eifel, &c. |
| 34 | Griffithides sp.? | | ........ | ........ | * | Button-mould Knob. | |
| | POLYPARIA. | | | | | | |
| 35 | Favosites fibrosa ........ | Goldf.—Sil. Syst., p. 682, pl. 15, fig. 6 and 7. | * | * | ........ | Falls of Ohio, Columbus, O., New York. | Russia, Eifel. |

9*

A TABLE OF FOSSILS FOUND IN THE VICINITY OF LOUISVILLE IDENTICAL WITH EUROPEAN SPECIES.—CONTINUED.

| Number of Species | GENERA AND SPECIES. | AUTHORS AND REFERENCES. | FORMATIONS IN EUROPE. | | | LOCALITIES. | PRINCIPAL LOCALITIES IN EUROPE. |
|---|---|---|---|---|---|---|---|
| | | | Silurian. | Devon. | Carbonif | | |
| 36 | Favosites Gothlandica.... | Lam.—Sil. Syst., p. 682, pl. 15, fig. 314 | * | * | ........ | Falls of O , Charlestown landing, Tenn., N. Y. | England, Russia, Eifel, &c. |
| 37 | " spongites...... | Goldf.—Sil. Syst , p. 682, pl. 15, fig. 8 and 9. | * | * | ........ | Falls of Ohio, Tennessee. | England, Russia, Eifel, &c. |
| 38 | " polymorpha ... | Goldf.—Sil. Syst., p. 694, pl. 15, fig. 2. | * | * | ........ | Falls of Ohio, Clarke co., Ia., New York. | Eifel, England, &c. |
| 39 | " basaltica....... | De Blainville—Sil. Syst., p. 682. | * | * | ........ | Falls of Ohio. | Gothland, Eifel. |
| 40 | Sarcinula costata ........ | Goldf.— | * | ........ | ........ | Bear-Grass. | |
| 41 | Aulopora serpens......... | Goldf.—Sil. Syst , p. 675, pl. 15, fig. 5. | * | * | ........ | Falls of Ohio, Perry co., Tenn. | Gothland, Dudley, Eifel, &c |
| 42 | " tubaeformis.... | Goldf.—Sil. Syst., p. 676, pl. 15, fig. 8. | * | * | ........ | Falls of Ohio. | Wenlock, Eifel. |
| 43 | Catenipora escharoides... | Lam.—Sil. Syst., p. 685, pl. 15, fig. 4. | * | *? | ........ | Bullitt co., Ky., N. York, Bear-grass. | Eifel (very rare), Gottland, Aymestery. |
| 44 | Cystiphyllum vesiculosum | Phil. pal. foss , pl. 4, fig. 12. | ........ | * | ........ | Falls of Ohio. | Eifel, South Devon. |
| 45 | Syringopora ramulosa.... | Goldfuss. | ........ | ........ | * | Falls of Ohio, Floyd co , Ia. | Olne, Russia. |
| 46 | Retepora prisca.......... | Goldfuss. | ........ | * | ........ | Falls of Ohio. | England. |
| 47 | Cyathoxonia cornu...... | | ........ | ........ | * | Button-mould Knob. | |
| 48 | Stromatopora polymorpha | Goldf., pl. 8, fig. 5, Sil. Syst., pl. 15, fig. 31. | * | * | ........ | Bear-Grass, Falls of Ohio. | Wenlock, Dudley. |
| 49 | Lithostrotion floriforme... | Flemming. | ........ | ........ | * | Floyd co., Ia.; Hardin co., Ky. | England. |
| | CRINOIDEA. | | | | | | |
| 50 | Cyathocrinus geometricus | Goldf , pl. 8, fig. 5, Phil. pal. foss p. 60, fig 41. | ........ | * | ........ | Button-mould Knob. | Newtown, Eifel. |

## Figures and Tables

**Figure f1:**